# Microtopography screening to modulate the mitogenic effects of aqueous humor on human tenon fibroblasts

**DOI:** 10.3389/fbioe.2026.1854721

**Published:** 2026-06-19

**Authors:** Phani Krishna Sudarsanam, Nikita Konshin, Jan de Boer

**Affiliations:** Department of Biomedical Engineering, Institute for Complex Molecular Systems, Eindhoven University of Technology, Eindhoven, Netherlands

**Keywords:** aqueous humor, biomaterials, glaucoma, micro topography, proliferation

## Abstract

**Background:**

Glaucoma is a leading cause of blindness, with elevated intraocular pressure being a primary risk factor due to the accumulation of aqueous humor in the anterior chamber. Aqueous humor is known to modulate the proliferation of surrounding fibroblasts, which may influence the long-term success of glaucoma drainage devices. This study investigates whether the mitogenic effects of aqueous humor can be modulated through the control of implant surface microtopography.

**Methods:**

A biomaterial library comprising 32 distinct micro topographies was fabricated using the biocompatible polymer poly (styrene-block-isobutylene-block-styrene) (SIBS) via hot embossing. Bovine aqueous humor was utilized in cell proliferation assays to evaluate its effects on human Tenon fibroblasts, alongside an analysis of the cellular morphological response to different micro topographies.

**Results:**

Aqueous humor exhibited a dose-dependent effect on human Tenon fibroblasts proliferation, ranging from 0.1% to 50% concentration. Fibroblast proliferation was significantly influenced by surface topography, which in turn correlated with alterations in cell morphology. Certain topographies decreased the mitogenic effects of aqueous humor, whereas others enhanced cell proliferation in a synergistic manner (e.g., T-1153 vs. T-930).

**Conclusion:**

Our findings demonstrate that implant surface microtopography modulates aqueous humor-induced human Tenon fibroblast proliferation. These insights provide a potential strategy for optimizing the design of glaucoma drainage devices to regulate fibrotic responses and improve long-term device patency.

## 1 Introduction

Aqueous humor is a clear fluid synthesized by the ciliary body in the eye, playing a crucial role in nutrient transport, waste removal, and maintaining clear optical path (Goel et al., 2010; Cholkar et al., 2013). It also regulates intraocular pressure (IOP) within the anterior chamber typically around 14 mmHg. Elevated IOP can lead to glaucoma, a neurodegenerative condition that damages the optic nerve and causes irreversible vision loss. By 2040, an estimated 110 million individuals worldwide will be affected by glaucoma (Tham et al., 2014). IOP increases due to an imbalance in aqueous humor secretion and outflow, with the trabecular meshwork accounting for 50%–90% of outflow (Weinreb et al., 2014). To manage IOP, glaucoma drainage devices (GDDs) have been developed to drain aqueous humor from the anterior chamber into a filtration bleb formed between Tenon’s capsule and the sclera. Devices such as the EX-PRESS glaucoma filtration device, Baerveldt implant, and Ahmed glaucoma valve are increasingly used due to their higher success rates compared to trabeculectomy (Ishida et al., 2021). However, their efficacy is often compromised by fibrotic encapsulation, which blocks drainage and leads to implant failure.

The success rate of GDDs depends on the bleb survival, and patients often require revision surgery due to eye inflammation and scarring. The failure of Baerveldt and Ahmed implants is primarily due to the thick fibrous capsule formed around the device, which inhibits the absorption of aqueous humor into the sub tenon’s space (Schaefer et al., 2015). Success rates vary across different models and patient groups, likely due to variations in aqueous humor composition (Siegner et al., 1995; Ceballos et al., 2002; Schlunck et al., 2016). Growth factors in aqueous humor infiltrate the bleb post-surgery, activating fibroblasts, which differentiate into myofibroblasts leading to excessive matrix deposition and stiff blebs. Jung et al. demonstrated that aqueous humor promotes fibroblast proliferation and collagen deposition, reinforcing its role in fibrotic bleb formation (Jung et al., 2013).

Aqueous humor composition is critical in human Tenon fibroblasts (HTFs) proliferation and differentiation, influencing bleb fibrosis and implant failure (Freedman and Iserovich, 2013; Nakamura-Shibasaki et al., 2013; Duvesh et al., 2017). Early studies revealed differential fibroblast proliferation in aqueous humor from glaucoma versus cataract patients (Herschler et al., 1980; Burke et al., 1982). Glaucoma associated aqueous humor contains a cytokine cocktail, including TNF-α, IL-6, IL-8, and MIP-1β, which trigger inflammation, while TGF-β1 and TGF-β2 drive fibroblast proliferation and differentiation (Du et al., 2016; Duvesh et al., 2017; Khalef et al., 2017). Bleb failure is correlated with elevated TGF-β2 levels, while patients with lower levels exhibit better bleb survival (Freedman, 2021). Additionally, high SPARC levels in aqueous humor are associated with increased surgical failure (Zhang et al., 2018). These cytokines, also present in normal aqueous humor, have been shown to induce fibrosis in animal models (Jung et al., 2013). An *in vitro* study demonstrated that exposure to TGF-β1 increased HTF contractility and α-SMA expression within 48 h at both gene and protein levels (Meyer-ter-Vehn et al., 2006).

As an alternative strategy to antimetabolite treatments that are prone to side effects, we propose modulating the mitogenic effect of aqueous humor using biomaterials that attenuate its biochemical signalling. Material properties such as stiffness, elasticity, and topography are known to influence cell adhesion and signalling (Berry et al., 2004; Liliensiek et al., 2006; Choritz et al., 2010; Gittens et al., 2011). One study investigated the use of micro actuators to mitigate biofouling in GDDs, demonstrating the potential of active microscale systems to improve long-term implant functionality by mechanically clearing protein accumulation within the drainage channels (Park et al., 2018). In parallel, several studies have highlighted the critical role of biomaterial surface properties in regulating cellular responses at the implant–tissue interface. Micro scale and nano scale topographies have been shown to influence cell adhesion, morphology, migration, proliferation, and differentiation through mechano-transduction signaling pathways. Previous work from our group demonstrated that algorithm-generated topographical libraries can systematically modulate cell fate decisions and cellular attachment behaviors (Unadkat et al., 2011; Sudarsanam et al., 2026). Specific topographies have been shown to synergize with TGF-β signalling, leading to increased expression of target genes (Vermeulen et al., 2020). This study aims to identify material properties that influence the mitogenic activity of aqueous humor using topographies from our TopoChip library (Zhao et al., 2017). In this manuscript, we investigate how surface modifications using micro topographies affect HTF proliferation in response to aqueous humor.

## 2 Materials and methods

### 2.1 Microtopography fabrication

The topographies used in this manuscript were selected from previous screen (Sudarsanam et al., 2026). A silica wafer with 32 selected topographies, each with a diameter of approximately 2 cm, was produced by deep reactive etching to create a negative mold. This mold was then used to make a positive PDMS mold and subsequently, a second negative mold was created using OrmoStamp (Micro Resist Technologies GmbH, Germany), which was used for transferring the topographies onto Poly (Styrene-block-Isobutylene-block-Styrene) (SIBS; Santen, FL, United States). The fabrication process of the topographies onto SIBS was carried out using hot embossing tool (Kaplan Scientific, Netherlands), where SIBS was melted at a temperature of 150 °C, and a pressure of 4 tons was applied for 15 min and demolded at 90 °C. All the fabricated topographies were plasma treated to make surface hydrophilic for cell experiments. Plasma treatment was done for 30 s at 50 W, 10 sccm bleeding with O_2_. The quality of the fabricated SIBS surfaces was tested using a Sensofar profilometer.

### 2.2 Aqueous humor collection

Bovine eyes were harvested from adult cows collected from a local slaughterhouse (Life Tec, Netherlands). The harvested bovine eyes were placed in cold PBS and kept at 4 °C and were used within 4–5 h after slaughter to acquire the aqueous humor. All the eyes were placed in a petri dish with cold sterile PBS (Gibco). A sterile syringe with a 30-gauge needle was inserted into the anterior chamber of the eye and the aqueous humor was retrieved using the syringe. The isolated aqueous humor samples were stored in a −80 °C freezer for long-term use. Approximately 12 donor eyes were used for aqueous humor isolation throughout the study. Importantly, aqueous humor samples from different donors were not pooled at any stage, and each experiment was performed using samples derived from individual donors.

### 2.3 Cell culture

Normal human dermal fibroblasts (HDF, Lonza) were thawed into two T-150 culture flasks (Thermo Fisher) at a seeding density of 2,000 cells/cm^2^ using Fibroblast growth medium-chemically defined (FGM-CD™) and after reaching 80% confluency, the two flasks were trypsinized and sub cultured into four new T-150 flasks till they reach confluency and at passage 3, all the cells were frozen for future use. For later passages, HDFs were cultured using DMEM (Gibco) medium with 10% (v/v) fetal bovine serum (FBS; Sigma) and Penicillin/Streptomycin (100 U/ml; Gibco).

HTFs used in this study were isolated from primary human Tenon tissue samples from glaucoma patients at the University Eye Clinic in Maastricht with permission from the local ethical committee (permission number 2019–0,983). All the cells used in this study were from a single donor. Basic HTF medium consisted of Advanced DMEM with GlutaMAX (Gibco). Basic medium was supplemented with 10% (v/v) fetal bovine serum (FBS; Gibco), Penicillin/Streptomycin (100 U/ml; Gibco) along with 0.2 mM of L-Glutamine (Gibco). The initial passage of the cells isolated was supplemented with 20% FBS till passage 2. Cells were grown at 37 °C in a humid atmosphere at 5% CO2. For all the experiments, both HDFs and HTFs cells at passage 5-6 were used.

### 2.4 Proliferation assay

HTFs were seeded at 20,000 cells/cm^2^ in basic HTF medium without FBS on a flat 96-well plate (Greiner Bio) and incubated at 37 °C in a humid atmosphere at 5% CO_2_ for 24 h. The next day, the cells were washed with PBS and treated with varying dilutions of aqueous humor in basic HTF medium added with 10 μM of EdU (Invitrogen). Cells treated with 10 μg/ml of TGF-β1 (Peprotech) were used as a positive control. The well plate was then further incubated for 24 h at 37 °C in a humid atmosphere at 5% CO_2_ after which cells were fixed with 3.7% (v/v) paraformaldehyde (Sigma- Aldrich).

### 2.5 Metabolic assay

HTFs were seeded in triplicate at 10,000 cells/cm^2^ in basic HTF medium in a 96-well plate with different dilutions of aqueous humor and incubated at 37 °C for 48 h. After 48 h, the cells were washed with sterile PBS, and 90 μl of DMEM medium without any phenol red (Gibco) was added and placed back in the incubator for 1 hour after which 10 μl Presto Blue solution (Invitrogen) was added. Cells were kept in the incubator for 3 h after which the fluorescence was measured at excitation/emission wavelengths of 530 nm/590 nm with a plate reader (Tecan). In this study, a blank control was measured with DMEM without phenol red and presto blue mixture.

### 2.6 Topography screening

Following hot embossing, the SIBS topographies were punched into circular samples corresponding to the diameter of a 24-well plate using a metal biopsy puncher, resulting in a surface area of 1.9 cm^2^ per sample. Individual topographies were placed into separate wells of a 24-well plate and secured at the bottom of the wells using O-rings. The well plates were sterilized under UV light in a biosafety cabinet for 15 min, followed by three washes with 70% ethanol. After sterilization, cells were seeded onto the topographies at a density of 10,000 cells/cm^2^. The cells were subsequently serum-starved 24 h prior to EdU staining, as described in the section below.

### 2.7 Staining and imaging

Staining was performed using the Click iT™ EdU cell proliferation kit with Alexa Fluor™ 647 dye according to the vendor’s protocol (Thermofisher). In brief, the fixed cells were permeabilized and washed with 3% bovine serum albumin (BSA, Merck) three times at room temperature and then the EdU reaction cocktail containing reaction buffer and additive mixed with Alexs Flour azide that binds to the EdU was added. For F-actin and α-SMA staining, the cells were permeabilized with 0.5% Triton X-100 (Merck) dissolved in PBS for 10 min and blocked with 3% (w/v) bovine serum albumin (Merck) in PBS for 30 min and then with 5% (v/v) goat serum (Fisher Scientific) dissolved in PBS for 30 min. Next, cells were incubated with a primary antibody using mouse monoclonal anti α-SMA diluted in PBS (1:600; Sigma: A5228) overnight at 4 °C. Next day, cells were washed 3 times with PBS and then incubated with a secondary antibody goat anti-mouse Alexa Flour 488 diluted in PBS (1:500; Molecular probes: A21121) and incubated in the dark for 1 h at room temperature. For the α-SMA screen, cells were washed three times with PBS and stained with phalloidin-TRITC diluted in PBS (1:200, ThermoFisher) for 45 min and washed 3 times again with PBS and stained with 4′,6-Diamidino-2-Phenylindole (DAPI) (Sigma Aldrich; 1:500) for 15 min. All the stained samples were imaged using Nikon Ti2 high content imaging microscope. For the proliferation experiments to test the role of bovine aqueous humor on HTFs and HDFs, three images were acquired from each of three technical replicates for all the conditions in all biological replicates and the means of each biological replicate for each condition were plotted in the graph. For screening experiments, three images were acquired from each triplicate in topographical library and EdU percentage for individual images was measured. Means of each image were plotted in the bar plots. Percentage of EdU positive cells for each image was calculated and the plots represent mean values of the all the images from each replicate.

### 2.8 Image and data analysis

Open-source software Cell Profiler 4.2.1 (CP) was used for image analysis. To perform automated image analysis in CP, a robust pipeline able to recognize different cell features was built. A custom pipeline was built where the background was corrected using illumination correction for the images, nuclei number was measured using the primary objects module. This measures both total cells and EdU positive cells creating Otsu thresholding and gating the size for the objects within the range of 10–45 pixels. For morphological fingerprinting of the cell cytoskeleton, we used global minimum cross-entropy thresholding with the propagating algorithm to identify cell outlines. For our Cell Profiler pipelines, please visit https://github.com/cbite/Aqueoushumor.

### 2.9 Statistical analysis

To find statistical significance between two conditions to find the role of aqueous humor on HDFs and HTFs and for the screening experiments, we performed one-way ANOVA to compare multiple groups to find significance. Pearson correlation analysis was performed to assess associations between topographical design descriptors and HTF proliferation. For each selected topography, the percentage of EdU-positive cells was calculated from replicate images and average per topography. These mean percentage of aqueous humor treated EdU-positive cells were then matched to the corresponding topographical design descriptors. Pearson’s correlation coefficients were calculated in Python using the panda’s package to identify descriptors associated with increased or decreased proliferative response. Descriptors with high absolute correlation coefficients were considered candidate topographical parameters associated with EdU incorporation.

## 3 Results

### 3.1 Bovine aqueous humor induces proliferation of human tenon fibroblasts

**FIGURE 1:**
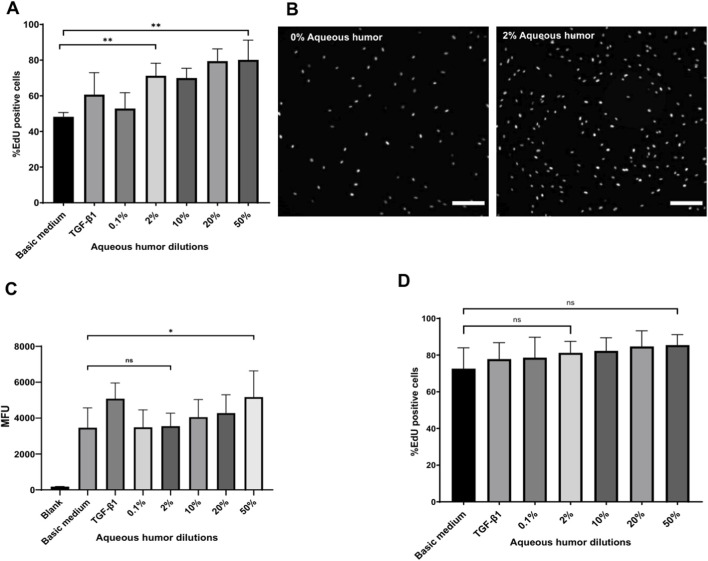
Bovine aqueous humor induced cell proliferation in human Tenon fibroblasts. **(A)** Percentage of EdU positive HTFs exposed to dilutions of aqueous humor. (one-way Anova with Tukey’s multiple comparison test, **p < 0.005). **(B)** Grayscale images of HTFs stained for EdU treated with no aqueous humor and 2% aqueous humor. Scale bar: 200 µm. **(C)** HTF metabolic activity treated with different dilutions of aqueous humor. MFU: Mean fluorescent units were calculated. (one-way Anova with Tukey’s multiple comparison test, *p < 0.05). **(D)** Plot showing percentage of EdU positive cells of human dermal fibroblasts when exposed to different dilutions of aqueous humor. Error bars in all the plots represent standard deviation between the means of three biological replicates (n = 3).

To validate the mitogenic effect of aqueous humor, HTFs were serum starved to synchronize their cell cycle before exposure to varying concentrations of bovine aqueous humor. Cells were treated with bovine aqueous humor and EdU for 24 h, and proliferation was assessed via EdU staining. In control conditions (basic medium), 48% of cells were EdU-positive, increasing to 60% with TGF-β1. Notably, proliferation rose to 71% with 2% aqueous humor and up to 80% with 20% and 50% aqueous humor (Figure 1A). A significant increase in EdU-positive cells was observed with 2% aqueous humor compared to untreated samples (Figure 1B). Metabolic activity was also evaluated, showing an increase with TGF-β1 but not significantly in aqueous humor exposed cells, except at 50% (Figure 1C). Interestingly, HDFs treated under the same conditions showed no significant proliferation differences, including in the TGF-β1 control group (Figure 1D). These findings confirm that HTFs are uniquely responsive to the mitogenic effects of bovine aqueous humor. Based on these results, 2% of aqueous humor was selected for further studies.

### 3.2 Topographical modulation of HTF morphology affects the mitogenic capacity of aqueous humor

**FIGURE 2:**
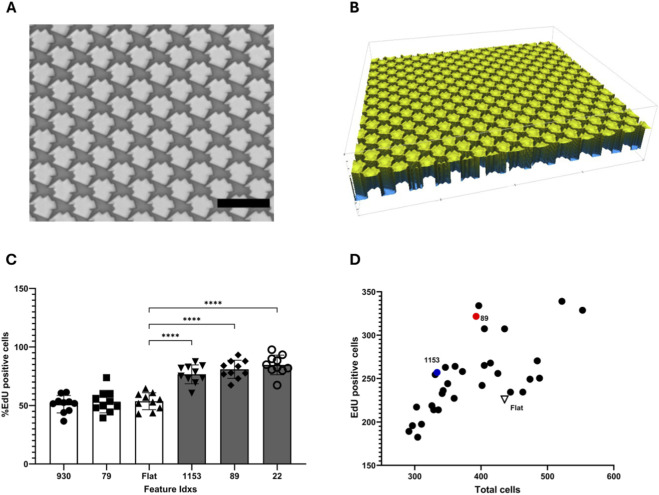
Topographies induced varied proliferative response of HTFs. **(A)** Bright field image of T-1153 demonstrates fidelity of topography production on SIBS polymer (Scale bar: 100 μm). **(B)** T-1153 imaged by profilometry to check the height profile. **(C)** Plot showing three topographies from the screen with high and low percentage of EdU positive HTFs after 48 h without aqueous humor. (Low %EdU; %High EdU, one-way Anova with Tukey’s multiple comparison test, ***p < 0.0001). Error bars represent the standard deviation from the EdU percentage calculated from all images from the duplicates (N = 2). **(D)** Scatter plot showing the number of EdU positive cells versus total number of cells on the topographies. Topographies T-89 (red) and T-1153 (blue) and flat surface are highlighted.

To assess whether surface topography influences HTF sensitivity to aqueous humor, 32 micro topographies were selected from a previous TopoChip screen based on their effects on HTF attachment. These topographies were fabricated using the SIBS polymer, commonly used in glaucoma filtration devices (Acosta et al., 2006) (Figure 2A) and verified via profilometry to check the height profiles (Figure 2B). HTF proliferation was first examined on SIBS micro topographies in the absence of aqueous humor. Cells were serum-starved for 24 h, then cultured in basic medium for 48 h. Interestingly, HTFs on a flat surface showed one of the lowest EdU-positive percentages (53%), contrasting with previous findings on polystyrene (Vasilevich et al., 2020) likely due to SIBS anti-fouling properties. Proliferation varied across micro topographies, from 51% (T-930) to 84% (T-22) (Figure 2C). Percentage of EdU-positive cells for all the 32 screened micro topographies is added in Supplementary Figure S1. A correlation was observed between low total cell numbers and low EdU-positive cell count, though at intermediate levels, this relationship was inconsistent (Figure 2D).

**FIGURE 3:**
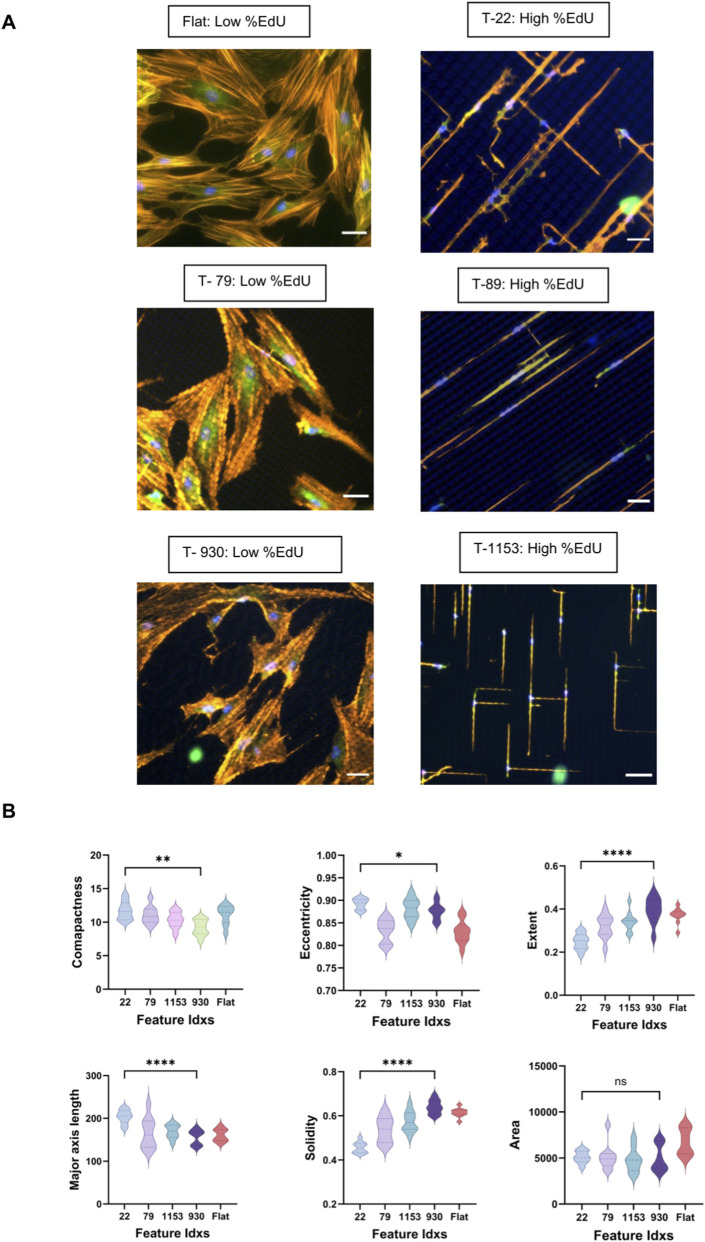
Fibroblast morphology changes when grown on topographies. **(A)** Fluorescent images showing the changes in cell shape of topographies flat, T-79, T-930 (Low %EdU) and T-22, T-89, T-1153 (High %EdU) (yellow; f-actin, blue; nuclei, green; α-SMA), Scale bar: 100 μm. **(B)** Different cellular shape parameters treated with aqueous humor (compactness, eccentricity, extent, major axis length, solidity, area) displayed using violin plots. Topographies T-930 (low %EdU), T-22 (High %EdU) exhibit distinct differences in morphological fingerprint in presence of aqueous humor. Mean of all individual objects in each image was measured and the means of each image per each topography from the duplicates were plotted (N = 2) of (one-way Anova with Tukey’s multiple comparison test, *p < 0.05, ***p < 0.0005, ****p < 0.0001, ns: not significant).

HTF morphology differed across micro topographies. High-proliferation surfaces (e.g., T-22, T-89, T-1153) confined cells within topographical structures, while low-proliferation surfaces (e.g., T-79, T-930, flat) allowed cells to spread (Figure 3A). The design of these micro topographies is shown in Supplementary Figure S2. A cell shape fingerprint analysis (compactness, eccentricity, extent, major axis length, solidity, area) revealed differences between high- and low-proliferation topographies, with significant variations in compactness, extent, and solidity but not cell area (Figure 3B). These findings demonstrate that surface topographies influence HTF morphology and proliferation.

### 3.3 Topographies influence aqueous humor mediated cell proliferation of fibroblasts

**FIGURE 4:**
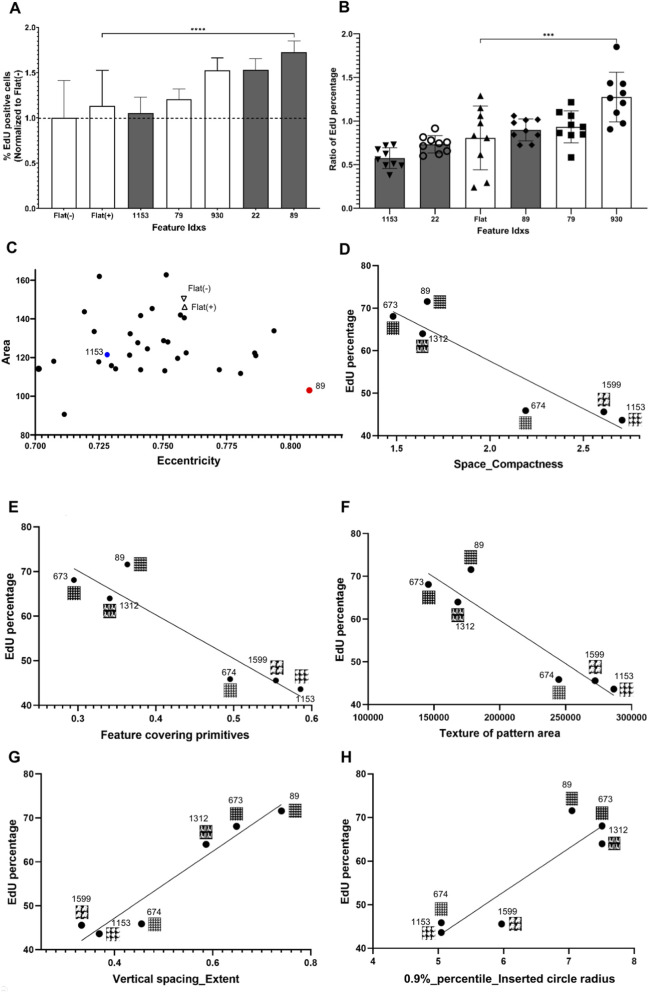
Modulation of aqueous humor-induced proliferation of HTFs by topographies. **(A)** Percentage of EdU positive HTFs treated with bovine aqueous humor for 24 h for the selected topographies from the screen. One-way Anova with Tukey’s multiple comparison test, ****p < 0.0001). **(B)** Ratio of EdU percentage in aqueous humor treated versus untreated samples. (one-way Anova with Tukey’s multiple comparison test, ***p < 0.0005). (Low %EdU; %High EdU represent the EdU percentage from no aqueous humor treatment) **(C)** Scatter plot of nuclear shape features area and eccentricity. **(D–H)** Pearson correlation plots with high EdU percentage and low EdU percentage topographies treated with aqueous humor show the top TDDs that had positive and negative correlations between each other.

To examine if the mitogenic effect of aqueous humor can be modulated, an EdU assay was performed on the same 32 micro topographies in the presence of aqueous humor. A 7% increase in EdU-positive cells on flat SIBS polymer confirmed HTF sensitivity to aqueous humor-induced proliferation compared to that of Flat (−) which is a negative control with %EdU-positive cells untreated with aqueous humor (Figure 4A). Notably, T-89 showed a 26% increase compared to the flat surface, while T-1153 had the lowest EdU percentage despite its high proliferation in the absence of aqueous humor. However, no micro topography showed lower proliferation than the flat surface under aqueous humor treatment (shown in Supplementary Figure S3). To quantify topography influence, the EdU percentage ratio between aqueous humor treated and untreated conditions was calculated by dividing the %EdU of cells treated with untreated samples for each condition except for Flat (+) which was used as Flat control. For Flat (+), the ratio was calculated with treated to untreated samples. Although exposure times differed (48 h untreated vs. 24 h treated), the relative difference highlighted that T-1153 altered the aqueous humor-induced proliferation, while T-930 showed the highest fold change, indicating a synergistic effect (Figure 4B). Difference in EdU ratio for all the micro topographies shown in Supplementary Figure S4.

Given the variations in EdU-positive cells, nuclear shape differences were examined. Phalloidin staining was incompatible with EdU labelling, so only nuclear morphology was analyzed. While no major changes were observed on the flat surface, T-89 and T-1153 showed distinct nuclear shapes. T-89, which had the highest proliferation, exhibited elongated nuclei confined within topographical structures, whereas T-1153, with the lowest proliferation, had more circular nuclei (Figure 4C). These results demonstrate that topography influences aqueous humor-mediated HTF proliferation and nuclear morphology.

### 3.4 Top topographical design parameters affect aqueous humor mediated cell proliferation

Pearson correlation analysis was conducted to assess the relationship between topographical design descriptors (TDDs) and changes in EdU incorporation following aqueous humor treatment. This analysis aimed to identify correlations between specific topographical features and the proliferation response of HTFs. Correlations were calculated by comparing the mean EdU percentage of the top three and bottom three proliferative topographies with the corresponding TDDs (added in Git Hub page from methods section). This was done to ensure clearer interpretability of feature–response relationships and to identify the most discriminative surface characteristics driving the observed biological differences. Several TDDs exhibited strong positive or negative correlations (r > 0.9) with HTF proliferation. Notably, the feature covering primitives, defined as the total area occupied by topographical structures, showed an inverse relationship with proliferation, with lower values associated with higher EdU percentages and *vice versa*. A similar trend was observed for other TDDs, including texture of pattern area, which quantifies surface roughness or smoothness, and compactness of feature spacing, which measures inter-feature distances based on pixel shape and distribution. (Figures 4D–F).

Conversely, positive correlations were identified for TDDs with low values of inserted circle radius and extent of vertical spacing-both of which describe the spatial arrangement of features. The inserted circle radius quantifies feature separation, while the extent of vertical spacing defines the distribution of unoccupied areas within a bounding box (Figures 4G,H). These findings suggest that specific topographical parameters directly influence HTF proliferation in response to aqueous humor, highlighting the role of surface microarchitecture in modulating cellular behavior.

## 4 Discussion

This study investigated whether micro topographical biomaterial modifications influence the mitogenic effect of bovine aqueous humor on HTFs. Previous research has shown that aqueous humor from rabbits and humans promotes HTF proliferation and migration at surgical sites (Herschler et al., 1980; Burke et al., 1982; Kottler et al., 2005). Notably, aqueous humor from glaucoma patients had a stronger proliferative effect than that from cataract patients (Herschler et al., 1980). Similarly, Burke et al. reported increased DNA synthesis in HTFs exposed to rabbit and human aqueous humor, with 2% aqueous humor also inducing an elongated cell shape (Burke et al., 1982) underscoring its role in glaucoma surgery outcomes. These findings collectively emphasize the significant influence of aqueous humor on the outcomes of glaucoma surgery.

Bovine aqueous humor was used due to its availability, as human aqueous humor is limited. Despite species differences, Burke et al. demonstrated that aqueous humor, regardless of origin, influences HTF proliferation. Bovine aqueous humor contains growth factors and chemotactic agents that drive proliferation and migration, aligning with findings from human studies. While species specificity is a limitation, bovine aqueous humor remains a valuable tool for *in vitro* screenings. Future studies using larger quantities of human aqueous humor could provide further insights, particularly into variations between glaucoma and non-glaucoma patients, which may impact ocular pressure (Yin et al., 2023). Although patient variability was not within the scope of this research, it would be interesting to investigate how HTFs respond to glaucomatous aqueous humor on selected topographies.

Few studies have explored biomaterials and surface modifications to regulate HTF behavior in GDDs (Ayyala et al., 2000; Choritz et al., 2010; Amoozgar et al., 2017). Choritz et al., 2010. found that rougher commercial glaucoma implants had higher HTF adhesion and encapsulation, while smoother surfaces had lower failure rates (Choritz et al., 2010). However, that study did not consider aqueous humor. Our research employs an algorithm-based approach to design topographies that affect both cell attachment and proliferation (Unadkat et al., 2011). This study aimed to bridge existing gaps by analyzing micro topography effects in aqueous humor, revealing that surface modifications can either enhance or reduce HTF proliferation compared to flat substrates.

An intriguing observation was the low proliferation of HTFs on flat SIBS polymer surfaces. Most topographies supported higher proliferation, with low EdU topographies showing a spread-out morphology and abundant stress fibers, while high EdU topographies confined cells between pillars. This contrasts with earlier studies where flat polystyrene surfaces promoted the highest proliferation in mesenchymal stem cells and tenocytes (Vasilevich et al., 2020; Dede Eren et al., 2021). The difference likely arises from substrate chemistry, as SIBS is an elastomeric polymer with lower stiffness, known for reducing encapsulation *in vivo.* Surface properties modulated HTF sensitivity to aqueous humor. Topography T-930 exhibited comparatively highest EdU ratio, suggesting that aqueous humor-induced proliferation could be amplified by cell physiological changes on this surface. This synergistic effect between topography and biochemical signals aligns with previous findings where topographies enhanced TGF-β2 induced tenogenic differentiation of mesenchymal stem cells, which could be blocked by interfering with actin-related signalling (Vermeulen et al., 2020). Since aqueous humor contains various growth factors, a similar mechanism may regulate HTF behavior.

This study provides a preliminary understanding of the influence of aqueous humor on fibroblast proliferation and its potential interaction with topography-mediated mechanotransduction. However, bovine aqueous humor was used in this study, and although previous literature suggests that compositional differences between bovine and human aqueous humor may be limited, future studies should validate these findings using aqueous humor obtained from human donors. Additionally, HTFs from a single donor were used, which may not fully capture donor-to-donor biological variability. Another limitation is the use of different experimental timelines between conditions, which restricts direct quantitative comparison of proliferation responses. Future studies using matched timelines, potentially optimized through varying initial seeding densities, would help address this to compare using absolute EdU positive cells more rigorously. Furthermore, while the present findings suggest a potential correlation between aqueous humor exposure and mechanotransduction-mediated regulation, this relationship requires further validation. Finally, *ex vivo* studies using selected topographies in ocular tissue models would be valuable for identifying optimal design parameters under physiologically relevant conditions and for better representation of *in vivo* environment during the development of next-generation GDDs.

## 5 Conclusion

Aqueous humor exerts a strong mitogenic influence on HTFs. Our findings demonstrate that micro topographies modulate this proliferative response, highlighting the potential of surface characteristics in directing cell behavior. These insights pave the way for therapeutic strategies and biomaterial designs aimed at addressing aqueous humor-induced proliferation while promoting favorable cell responses in glaucoma surgery and related applications. Future investigations will be essential to translate these findings into clinically relevant interventions, optimizing biomaterial performance for improved surgical outcomes.

## Data Availability

The datasets presented in this study can be found in online repositories. The names of the repository/repositories and accession number(s) can be found below: https://data.4tu.nl/private_datasets/YcugSi7ChVbeH1lOJXaQ6tw8Y5k0WpAFYABpFjhqzjw.
