# Supplementary figures and images for: Microtopography screening to modulate the mitogenic effects of aqueous humor on human tenon fibroblasts
Source: Front Bioeng Biotechnol. 2026 Jun 19;14:1854721. doi: 10.3389/fbioe.2026.1854721 (PMC13327865; doi:10.3389/fbioe.2026.1854721)

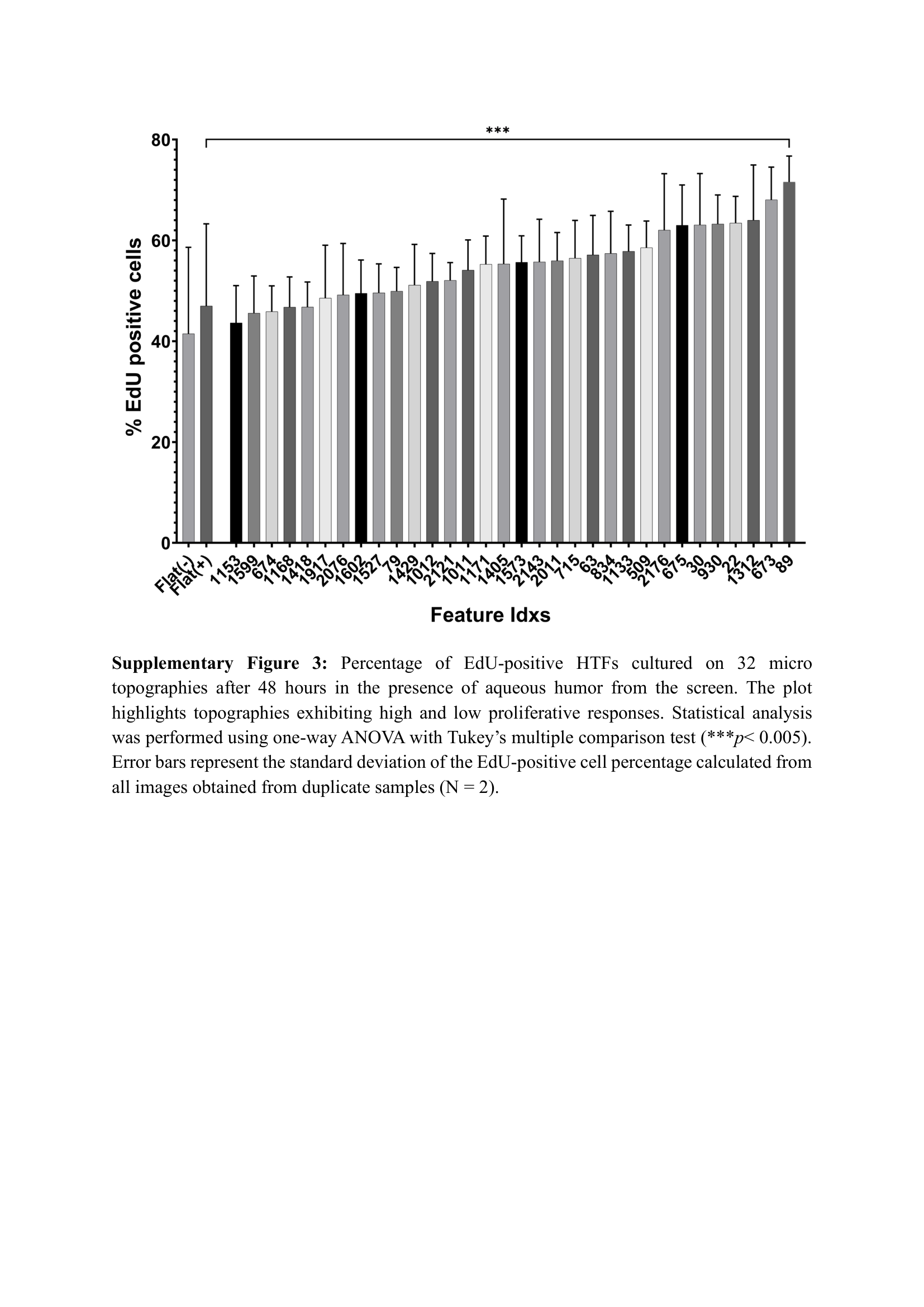

Supplement: Supplementary file 1 [file Image3.tiff]

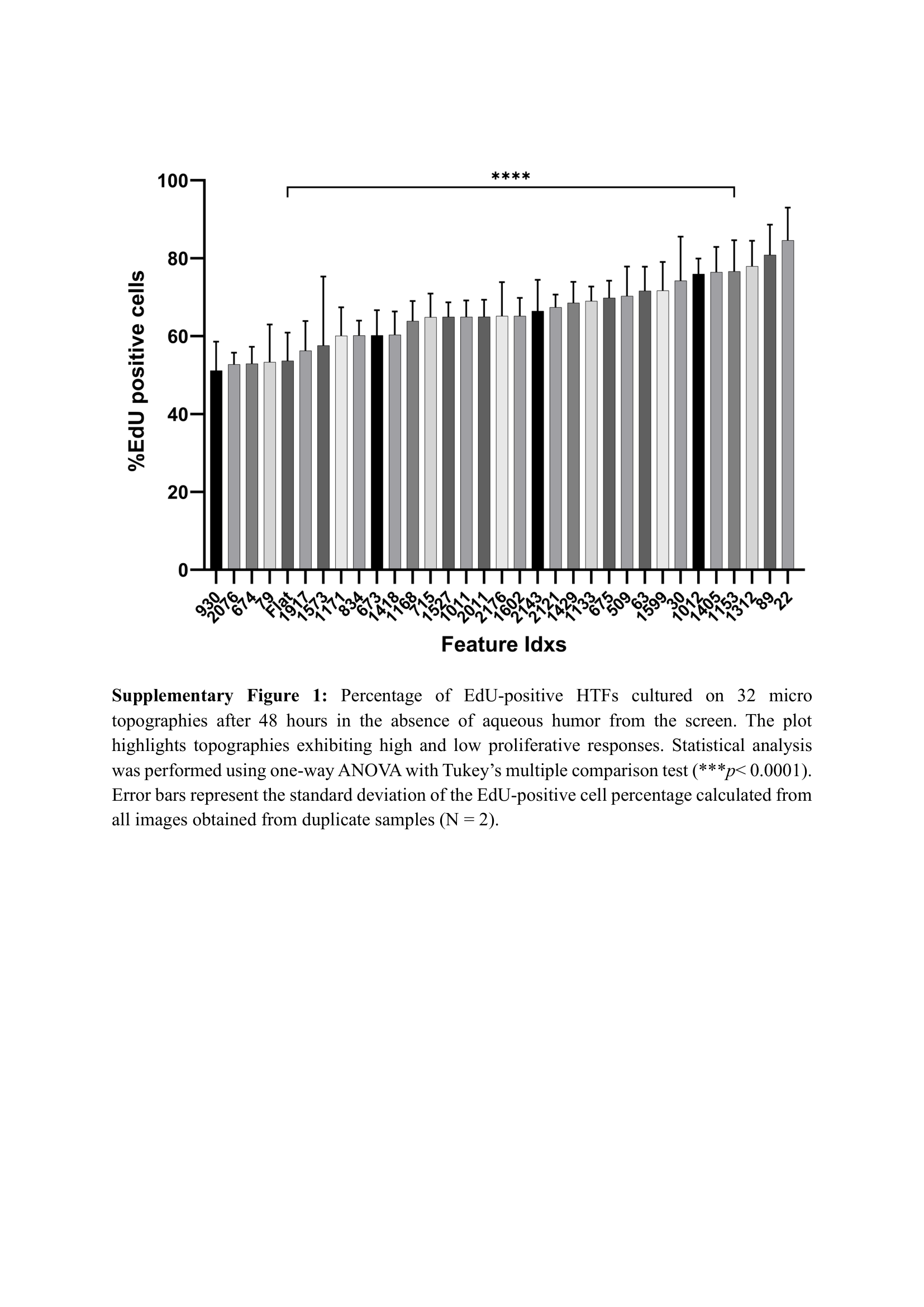

Supplement: Supplementary file 2 [file Image1.tiff]

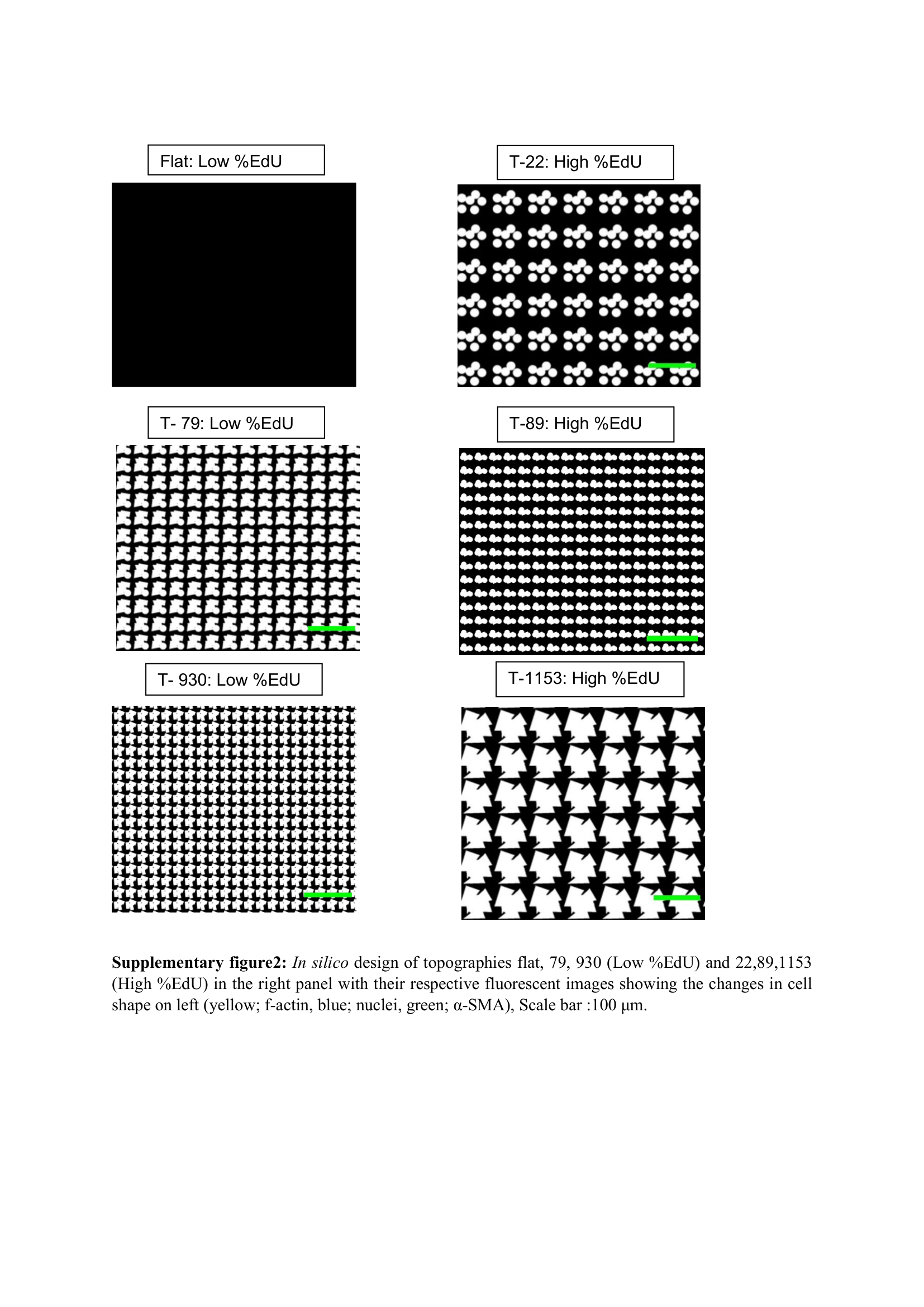

Supplement: Supplementary file 3 [file Image2.tiff]

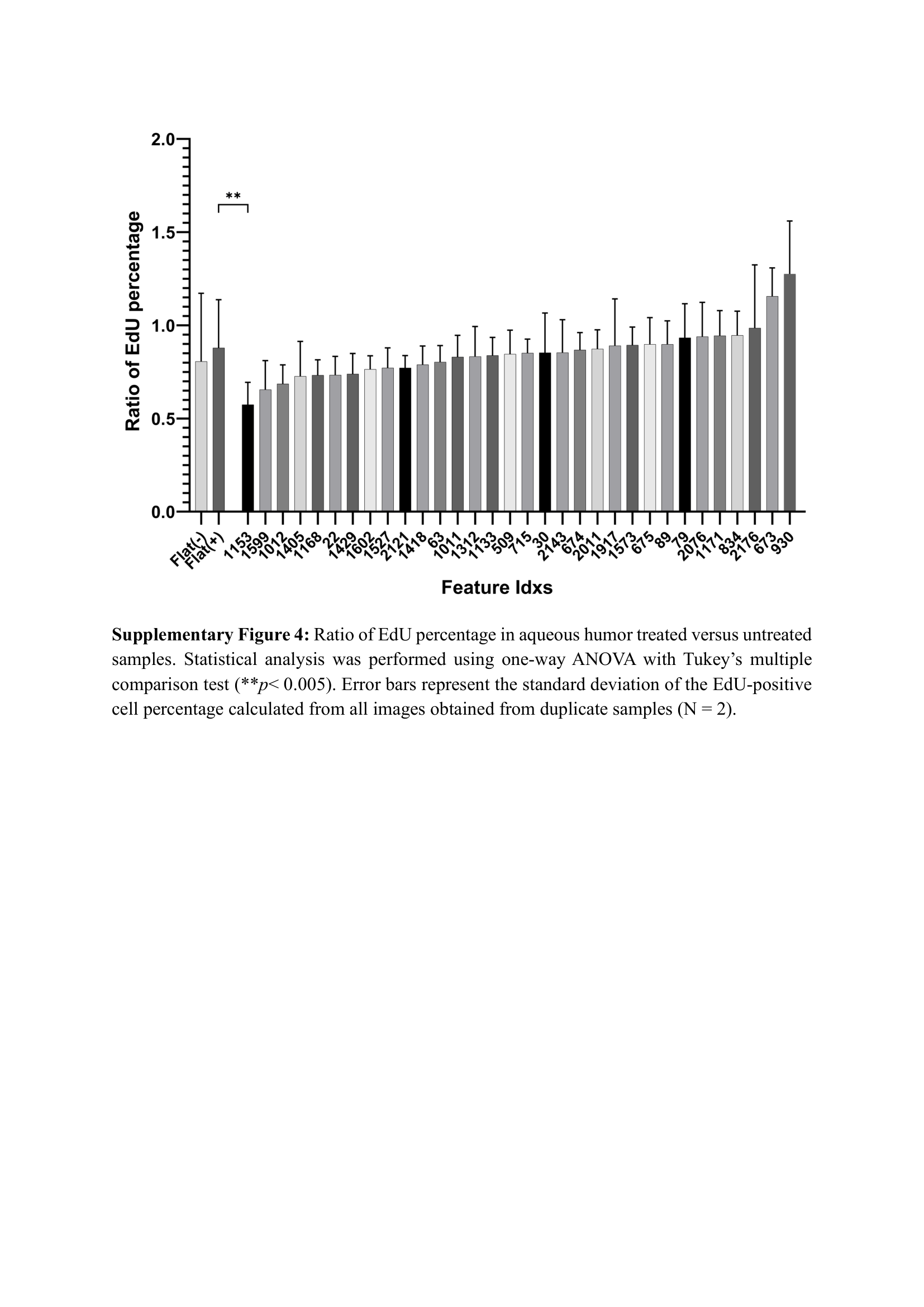

Supplement: Supplementary file 4 [file Image4.tiff]
